# Treatment as prevention for hepatitis C virus in the Middle East and North Africa: a modeling study

**DOI:** 10.3389/fpubh.2023.1187786

**Published:** 2023-07-13

**Authors:** Houssein H. Ayoub, Sarwat Mahmud, Hiam Chemaitelly, Laith J. Abu-Raddad

**Affiliations:** ^1^Mathematics Program, Department of Mathematics, Statistics, and Physics, College of Arts and Sciences, Qatar University, Doha, Qatar; ^2^Infectious Disease Epidemiology Group, Weill Cornell Medicine-Qatar, Cornell University, Doha, Qatar; ^3^World Health Organization Collaborating Centre for Disease Epidemiology Analytics on HIV/AIDS, Sexually Transmitted Infections, and Viral Hepatitis, Weill Cornell Medicine–Qatar, Cornell University, Qatar Foundation – Education City, Doha, Qatar; ^4^Department of Population Health Sciences, Weill Cornell Medicine, Cornell University, New York, NY, United States; ^5^Department of Public Health, College of Health Sciences, QU Health, Qatar University, Doha, Qatar; ^6^College of Health and Life Sciences, Hamad bin Khalifa University, Doha, Qatar

**Keywords:** Middle East and North Africa, hepatitis C virus, incidence, mathematical model, treatment as prevention

## Abstract

**Background:**

Direct-acting antivirals opened an opportunity for eliminating hepatitis C virus (HCV) infection in the Middle East and North Africa (MENA), the region most affected by HCV infection. Impact of HCV treatment as prevention (HCV-TasP) was investigated in 19 MENA countries.

**Methods:**

An age-structured mathematical model was used to assess program impact using epidemiologic and programming measures. The model was fitted to a database of systematically gathered HCV antibody prevalence data. Two main scenarios were investigated for the treatment roll-out to achieve (i) 80% reduction in HCV incidence by 2030, and (ii) incidence rate < 1 per 100,000 person-years by 2030.

**Results:**

In the target-80%-incidence-reduction scenario, number of treatments administrated by 2030 ranged from 2,610 in Lebanon to 180,416 in Sudan with a median of 53,079, and treatment coverage ranged between 40.2 and 78.4% with a median of 60.4%. By 2030, prevalence of chronic infection ranged between 0.0 and 0.3% with a median of 0.1%, and incidence rate, per 100,000 person-years, ranged between 0.9 and 16.3 with a median of 3.2. Program-attributed reduction in incidence rate ranged between 47.8 and 81.9% with a median of 68.5%, and number of averted infections ranged between 401 and 68,499 with a median of 8,703. Number of treatments needed to prevent one new infection ranged from 1.7 in Oman to 25.9 in Tunisia with a median of 6.5. In the target incidence rate  < 1 per 100,000 person-years scenario, number of treatments administrated by 2030 ranged from 3,470 in Lebanon to 211,912 in Sudan with a median of 54,479, and treatment coverage ranged between 55.5 and 95.9% with a median of 87.5%. By 2030, prevalence of chronic infection was less than 0.1%, and incidence rate, per 100,000 person-years, reached less than 1. Program-attributed reduction in incidence rate ranged between 61.0 and 97.5% with a median of 90.7%, and number of averted infections ranged between 559 and 104,315 with a median of 12,158. Number of treatments needed to prevent one new infection ranged from 1.3 in Oman to 25.9 in Tunisia with a median of 5.5.

**Conclusion:**

HCV-TasP is an effective and indispensable prevention intervention to control MENA’s HCV epidemic and to achieve elimination by 2030.

## Introduction

The Global Burden of Disease Study identified viral hepatitis as the 7th leading cause of mortality globally (1). Nearly half of this mortality is attributed to hepatitis C virus (HCV) (1), a blood-borne virus whose transmission is largely preventable (2, 3). Infection with HCV can cause acute hepatitis, fibrosis, cirrhosis, and liver cancer among other disease sequelae (4, 5).

Of all regions, the Middle East and North Africa (MENA) is the region most affected by HCV infection (1, 6–8). In 2019, an estimated 470,000 new HCV infections occurred in MENA (8), accounting for 30% of the global number of new HCV infections (8). Furthermore, in 2019, HCV caused 13,705 deaths due to liver cancer and 57,994 deaths due to cirrhosis and other chronic liver diseases in MENA (9).

Breakthroughs in HCV treatment, namely development of the highly efficacious direct acting antivirals (DAAs), has ushered a revolution in treating and controlling HCV infection, by reducing the burden and cost of managing liver-related conditions and potentially eliminating HCV infection as a public health threat (10–12). With DAAs availability and recent affordability, even in resource limited countries, the World Health Organization (WHO) called for ambitious global targets for diagnosis, treatment, and cure of viral hepatitis, signaling a major momentum towards HCV elimination by 2030 (13–15).

We assessed in this study the epidemiological impact of HCV treatment as prevention (TasP) in MENA countries, building on our previous work investigating impact of HCV-TasP in Egypt and Pakistan, the two countries most affected by this infection in MENA (16, 17), and following the success of this concept of TasP for HIV infection with a global impact (18). A dynamic-forecasting mathematical model applied individually to each country was used to (1) assess the treatment roll-out that is required to achieve 80% reduction in HCV incidence by 2030, as per the earlier WHO global target (13, 14), (2) assess the treatment roll-out that is required to achieve an incidence ≤5 per 100,000 people per year by 2030, as per the recently stipulated WHO global target (15), and (3) assess the treatment roll-out that is required to achieve an incidence rate < 1 per 100,000 person-years by 2030. The overarching goal of this study is to provide countries with the evidence-base necessary to plan and allocate resources to attend to the WHO global target of eliminating HCV as a public health threat by 2030 (13–15). This study, along with our previous research on the impact of HCV-TasP in Egypt and Pakistan (16, 17), provides modeling estimates for 21 out of the 24 countries in the MENA region, covering approximately 88% of the countries in the region.

## Materials and methods

### Mathematical model

An age-structured mathematical model was used to describe the dynamics of HCV transmission in a given total national population by adapting our previously published HCV-TasP models for Egypt and Pakistan (16, 17, 19), and factoring best practice for modeling guidelines (20) [Supplementary Material Figure S1 (SM)]. Further information can be found in the SM. Detailed descriptions of the modeling framework and its parameterization have been reported previously (16, 17, 19).

Briefly, the model consists of a set of coupled nonlinear differential equations that stratifies the population by age, HCV status, stage of infection, and level of risk of exposure to the infection. HCV natural history in the model was divided into three stages: primary acute infection, secondary acute infection, and chronic infection. The model assumed that the proportion of individuals who clear their primary acute HCV infection spontaneously is 25%, based on direct measurement from a prospective cohort study (21), and as informed by analysis of HCV viremic rates in MENA (22).

The model disaggregated the population into three age groups: children (1–14 years old), adults (15–59 years old), and older adult population (≥60 years old). To address the variation in exposure risk (23, 24), the model incorporated five distinct risk groups, each representing a different level of likelihood for exposure to HCV infection (with one being the lowest risk and five being the highest risk). Each risk group collectively encompassed different transmission pathways that share a similar level of infection risk.

In this mathematical modeling approach, explicitly considering the precise population composition or specific modes of transmission within each risk group poses a challenge. Instead of focusing on modeling specific modes of transmission, this approach aims to model different levels of risk of exposure to the infection within the population. Each risk group collectively represents various transmission pathways that share a similar level of risk for infection exposure. As an illustrative example, the lowest, middle, and highest risk groups could encompass the general population with minimal exposure risk (23–25), individuals who have undergone multiple transfusions, surgeries, or are on hemodialysis (23–25), and individuals who inject drugs and those undergoing high-risk medical procedures (23, 26), respectively. This categorization allows for a representation of the varying levels of exposure risk that exists within a population. Further details are found in earlier publications (16, 17, 19).

Individuals from different risk and age groups mixed in the model according to mixing matrices that included both an assortative component and a proportionate component (27, 28). The force of infection was expressed in terms of the effective contact rates, HCV transmission probability per contact, and mixing among the different age and risk groups.

### Data sources and model fitting

The model was parameterized using representative data for HCV transmission and natural history (Supplementary Table S1 in SM). The model was applied to 19 MENA countries that had minimally sufficient time series data for HCV antibody (Ab) prevalence to be able to apply the model and to fit it to these data. These countries included Afghanistan, Algeria, Iran, Iraq, Jordan, Kuwait, Lebanon, Libya, Morocco, Oman, Palestine, Qatar, Saudi Arabia, Somalia, Sudan, Syria, Tunisia, United Arab Emirates, and Yemen. The countries excluded from our analysis due to lack of sufficient time series data were Bahrain, Djibouti, and Mauritania. Furthermore, Egypt and Pakistan were also excluded as they had already been investigated in our previous work (16, 17). Demographics such as total population size and its past and future projections were obtained from the 2022 update of the database of the Population Division of the United Nations Department of Economic and Social Affair (29). The model for each country was fitted to reproduce these demographic projections for each included country.

The source of the HCV Ab prevalence data was the MENA HCV Synthesis Project Database (7), a compilation of published and unpublished epidemiological measures for HCV and related indicators in this region since first identification of this virus in 1989, based on a series of published systematic reviews that included all MENA countries (30–39). The Synthesis Project Database includes 2,621 Ab prevalence measures on 49,824,108 tested individuals. These measures were based on different at-risk population categories including general populations, blood donors, populations at intermediate risk, mixed populations, populations with liver related conditions, special clinical populations, populations at high risk, and people who inject drugs.

The number of Ab prevalence measures varied among the 19 MENA countries, ranging from 15 measures in Algeria and Kuwait to 477 measures in Iran. The median number of measures was 51. When considering the combined data for all 19 countries, the sample sizes of Ab prevalence measures ranged from 20 to 4,538,346, with a median of 307. The total sample size of Ab prevalence measures for each country ranged from 13,589 in Sudan to 20,693,801 in Saudi Arabia, with a median of 290,691.

The HCV Ab prevalence measures for each country were converted into a corresponding Ab prevalence time series by multiplying each measure in each specific population category by a category-specific anchoring factor. The model-generated temporal evolution of Ab prevalence was produced by fitting the time series of the anchored Ab prevalence measures and each country’s past and future demographics. The model fitting to each HCV Ab prevalence measure was weighted by the measure’s sample size.

The anchoring factor for the general population was set at 1 and the anchoring factor for blood donors was set at 1.72 (95% confidence interval (CI): 1.50–1.97) on the basis of meta-regression findings for the MENA region (25). The anchoring factor for each other population category was set by the model fitting. The Ab prevalence was also fitted to the pooled mean general-population Ab prevalence for each country on the basis of meta-analyses covering all MENA countries (30–39). This pooled mean Ab prevalence was set to represent Ab prevalence in the year 2010, as a median year for availability of data based on distribution of Ab prevalence data over time (24), and had a 2-fold higher weight in the fitting compared to the individual-study Ab prevalence data. Accordingly, the general population Ab prevalence data and the pooled Ab prevalence were used to fit the level and trend of Ab prevalence in each country while the Ab prevalence data for the other population categories were used to fit only the trend in Ab prevalence.

For Algeria, Kuwait, Morocco, Oman, Qatar, Somalia, Sudan, Syria, and Yemen, there were too few Ab prevalence data in populations other than the general population and blood donors, thus affecting the capacity of the model to fit the trend in Ab prevalence. Accordingly, the trend in Ab prevalence was also fitted to the overall trend in Ab prevalence data in all of MENA with a 2-fold higher weight in the fitting compared to the individual-study Ab prevalence data. The overall MENA trend was set at an average relative decline of 3% (95% CI: 2–4%) per year based on a recent meta-regression of all Ab prevalence data in the general population in MENA (24).

The model was fitted to HCV Ab prevalence trend data using a nonlinear, least-square fitting method. This technique was implemented in MATLAB® (40) using the Nelder–Mead simplex algorithm (41). To account for temporal variation in HCV Ab prevalence, temporal changes in risk of exposure to the infection were incorporated. This was parameterized through a Wood-Saxon function (16, 42, 43). This function is mathematically designed to describe and characterize transitions in terms of their scale or strength, smoothness or abruptness, thickness (duration), and the turning point (16, 28, 42, 43). Further details on the model fitting approach have been reported previously (16, 17, 19).

### Epidemiologic and programming measures

To inform public health response and following the previously published approach for Egypt and Pakistan (16, 17), we used epidemiologic and program indicators to assess the impact of the DAA treatment program in each country and in the 19 MENA countries combined. The impact was assessed for the total population of each country. The definitions of these indicators can be found in Table 1. These indicators are the outcomes of the model simulations. The year 2010 was chosen as a reference year for comparison of outcomes for consistency with the approach for Egypt and Pakistan (16, 17).

**Table 1:** Epidemiologic and programming measures used to quantify the impact of the HCV treatment program in each country, following our approach for Egypt and Pakistan (16, 17, 19).

Measure	Definition
HCV chronic infection prevalence	Proportion of a given population that are HCV antibody positive and RNA positive, that is, proportion of the population that are chronically infected with HCV. This also known as HCV viremic prevalence.
HCV incidence (also referred to as number of new HCV infections per year)	Number of new HCV infections in the total population over a specific timeframe such as a year.
HCV incidence rate per 100,000 person-years	Number of new HCV infections per person-time of the at-risk population, that is, HCV incidence per unit time (say a year) times 100,000 divided by the population size of the susceptible population.
HCV incidence per 100,000 people per year	Number of new HCV infections per unit time (say a year) times 100,000 divided by the total population size, that is including both those susceptible and infected.
HCV chronic infection prevalence reduction	Relative difference between HCV chronic infection prevalence at a given time point in presence of the treatment intervention, and that in the no-intervention counter-factual scenario. This measure was used to disentangle HCV chronic infection prevalence reduction attributed strictly to the program from that due to “natural” epidemic course. Specifically, this measure is calculated using the following expression: $\mathrm{Chronic infection prevalence reduction}=100\times \left[1-\frac{\mathrm{chronic infection prevalence in presence of the treatment intervention}}{\mathrm{chronic infection prevalence in the no-intervention counter-factual scenario}}\right]$
Incidence reduction	Relative difference between incidence at a given time point and incidence in 2010. This measure was used to define the target-80%-incidence-reduction scenario by 2030. The year 2010 was chosen as a reference year for comparison of outcomes, as suggested earlier by the WHO (14, 44), and for consistency with the approach for Egypt and Pakistan (16, 17).
Incidence rate reduction	Relative difference between incidence rate at a given time point in presence of the treatment intervention, and that in the no-intervention counter-factual scenario. This measure was used to disentangle incidence rate reduction attributed strictly to the program from that due to “natural” epidemic course. Specifically, this measure is calculated using the following expression: $\mathrm{Incidence rate reduction}=100\times \left[1-\frac{\mathrm{incidence rate in presence of the treatment intervention}}{\mathrm{incidence rate in the no-intervention counter-factual scenario}}\right]$
Number of averted infections	Difference between incidence after treatment program implementation, and that in the no-intervention counter-factual scenario. An annual discount rate of 3% was applied on future savings (that is infections averted).
Proportion of infections averted	Number of infections averted by the treatment program divided by the total number of new infections in the no-intervention counter-factual scenario within the same timeframe.
Number of treatments required to avert one new infection	Cumulative number of treatments divided by number of averted infections by the treatment program over a chosen timeframe.
Program treatment coverage	Number of living treated persons at a given time point divided by number of living chronically infected persons at that time point.

These measures are the outcomes of the model simulations.

HCV, hepatitis C virus; RNA, ribonucleic acid; WHO, World Health Organization.

### Treatment program scenarios

The treatment program was implemented in the model between 2023 and 2030 with the goal of achieving elimination by the 2030 target year. The program involved providing treatment at a specific rate for each country, ensuring a fixed probability per unit time for individuals to receive treatment. Eligibility for treatment was extended to all individuals aged 15 years or older who were chronically infected, regardless of the stage of liver disease. There was no prioritization of specific groups, and every eligible person had an equal chance of receiving treatment. The effectiveness of the treatment in real-world conditions was assumed to be 90% across all age and risk groups (45).

Three program scenarios were investigated for each country. The first one is the target-80%-incidence-reduction scenario where the annual treatment rate in each country is set at a level that can achieve 80% incidence reduction by 2030. The second one is the target-incidence- ≤ 5-per-100,000-people scenario where the annual treatment rate is set at a level that can yield an incidence ≤5 per 100,000 people per year in 2030. The third one is the target-incidence-rate- < 1-per-100,000-person-years scenario where the annual treatment rate is set at a level that can yield an incidence rate of <1 per 100,000 person-years in 2030. The latter scenario is the most ambitious scenario explored here and would lead to virtual elimination of HCV infection, per a conventional definition of infection elimination (46).

The second scenario was not a primary focus of our analysis, because several countries have reached or would reach an incidence ≤5 per 100,000 people per year by 2030 without scale-up of treatment (Supplementary Table S3). In some countries, current incidence rate may not be reflective of current prevalence of infection, as prevalence reflects incidence rate at earlier times when incidence rate was high. Therefore, we opted in this study to highlight a more ambitious elimination target for this infection.

### Uncertainty analysis

Uncertainty analyses were conducted to estimate the range of uncertainty around the epidemic projections and treatment program outcomes in each country. This was done by using the lower and upper bounds of the 95% confidence interval (CI) of each HCV Ab prevalence measure to generate, respectively, the highest and lowest model-fitted epidemic projections. The 95% CI of each HCV Ab measure was calculated in Matlab using the Clopper-Pearson method. The highest and lowest projections were used to estimate the 95% uncertainty interval (95% UI) of the impact of the program. Point estimates and associated 95% UIs were reported for each of the epidemiologic and programming indicators for each country.

## Results

Table 2 shows the impact of the treatment program scenarios on HCV chronic infection prevalence in the total population of each of the 19 included countries. Prevalence of chronic infection in 2022 (before the start of the program) ranged between 0.1% in several countries such as Algeria and Lebanon, and 1.0% in the United Arab Emirates, with a median of 0.3%. In absence of the treatment program, chronic infection prevalence was projected to decline in all 19 countries, but at different rates ranging from very slow decline to somewhat rapid decline. Prevalence of chronic infection by 2030 ranged between 0.1 and 0.9% across countries with a median of 0.2%.

**Table 2:** Impact of the treatment program scenarios on HCV chronic infection prevalence in 19 countries in the Middle East and North Africa.

Countries	HCV chronic infection prevalence in 2022 (%)	HCV chronic infection prevalence by 2030 (%)			HCV chronic infection prevalence reduction strictly attributed to the treatment program scenarios by 2030 (%)	
		No-treatment intervention scenario	Target 80% incidence reduction scenario^*^	Target incidence rate < 1 per 100,000 person-years scenario	Target 80% incidence reduction scenario^*^	Target incidence rate < 1 per 100,000 person-years scenario
Afghanistan	0.41 (0.26–0.67)	0.36 (0.21–0.62)	0.08 (0.05–0.11)	0.01 (0.01–0.01)	78.6 (75.7–81.3)	97.6 (95.3–98.8)
Algeria	0.10 (0.02–0.49)	0.08 (0.01–0.42)	0.03 (0.00–0.11)	0.01 (0.00–0.01)	67.5 (61.2–73.3)	86.4 (61.2–97.8)
Iran	0.15 (0.08–0.40)	0.12 (0.06–0.31)	0.05 (0.03–0.13)	0.03 (0.03–0.03)	54.5 (52.6–57.2)	74.5 (52.6–91.2)
Iraq	0.24 (0.10–0.63)	0.24 (0.08–0.52)	0.04 (0.02–0.06)	0.04 (0.02–0.04)	82.6 (81.0–88.5)	84.3 (81.0–97.8)
Jordan	0.16 (0.06–0.50)	0.14 (0.05–0.49)	0.02 (0.01–0.06)	0.02 (0.01–0.02)	83.4 (79.7–87.6)	83.8 (79.7–96.9)
Kuwait	0.84 (0.19–2.68)	0.63 (0.14–2.08)	0.32 (0.08–0.90)	0.02 (0.02–0.02)	48.2 (42.5–56.7)	97.0 (84.0–99.3)
Lebanon	0.11 (0.03–0.48)	0.06 (0.02–0.29)	0.03 (0.01–0.09)	0.02 (0.01–0.02)	53.9 (40.7–68.4)	70.2 (40.7–96.1)
Libya	0.94 (0.61–1.62)	0.89 (0.58–1.54)	0.23 (0.15–0.39)	0.05 (0.05–0.05)	74.0 (73.7–74.3)	94.6 (91.5–97.0)
Morocco	0.40 (0.22–0.70)	0.29 (0.16–0.52)	0.14 (0.08–0.24)	0.02 (0.02–0.02)	52.5 (50.8–54.6)	92.6 (86.4–95.7)
Oman	0.34 (0.18–0.63)	0.23 (0.12–0.45)	0.07 (0.04–0.13)	0.01 (0.01–0.01)	68.9 (66.1–71.8)	97.6 (95.1–98.8)
Palestine	0.31 (0.19–0.47)	0.23 (0.14–0.36)	0.06 (0.04–0.09)	0.06 (0.04–0.06)	74.3 (73.0–75.3)	74.3 (73.0–84.2)
Qatar	0.37 (0.37–0.44)	0.34 (0.34–0.41)	0.08 (0.07–0.09)	0.02 (0.02–0.02)	78.1 (78.1–78.5)	95.1 (95.1–95.9)
Saudi Arabia	0.40 (0.23–0.71)	0.29 (0.17–0.52)	0.15 (0.09–0.26)	0.03 (0.03–0.03)	48.1 (47.1–49.5)	91.0 (83.8–95.2)
Somalia	0.24 (0.08–0.66)	0.19 (0.05–0.56)	0.04 (0.01–0.08)	0.01 (0.01–0.01)	80.9 (73.9–86.1)	92.0 (73.9–98.0)
Sudan	0.43 (0.14–1.13)	0.35 (0.11–0.98)	0.08 (0.03–0.19)	0.02 (0.02–0.02)	77.8 (73.6–80.8)	93.2 (76.3–98.2)
Syria	0.29 (0.18–0.49)	0.24 (0.15–0.41)	0.06 (0.04–0.10)	0.06 (0.04–0.06)	74.8 (73.6–76.7)	74.8 (73.6–85.3)
Tunisia	0.30 (0.14–0.74)	0.23 (0.11–0.58)	0.09 (0.04–0.22)	0.09 (0.04–0.09)	61.0 (60.2–63.0)	61.2 (60.2–86.3)
United Arab Emirates	0.98 (0.52–1.87)	0.69 (0.36–1.39)	0.22 (0.12–0.44)	0.03 (0.03–0.03)	68.1 (66.5–71.5)	96.1 (92.6–98.2)
Yemen	0.60 (0.29–1.15)	0.39 (0.18–0.77)	0.18 (0.09–0.31)	0.02 (0.02–0.02)	54.8 (49.9–60.4)	95.8 (90.2–98.1)

^*^Incidence reduction was defined as the relative difference between incidence at a given time point and incidence in 2010. The year 2010 was chosen as a reference year for the target-80%-incidence-reduction scenario, as suggested earlier by the WHO (14, 44), and for consistency with the approach for Egypt and Pakistan (16, 17).

In presence of the treatment program, prevalence of chronic infection by 2030 ranged between 0.0 and 0.3% across countries with a median of 0.1% in the target-80%-incidence-reduction scenario and was less than 0.1% in the target incidence rate < 1 per 100,000 person-years scenario. The reduction in prevalence of chronic infection by 2030, strictly attributed to the treatment program, depended on the treatment coverage per each scenario in each country (Table 3). It ranged between 48.1 and 83.4% with a median of 68.9% in the target-80%-incidence-reduction scenario, and between 61.2 and 97.6% with a median of 91.0% in the target incidence rate < 1 per 100,000 person-years scenario.

**Table 3:** Key program indicators for the treatment program scenarios in 19 countries in the Middle East and North Africa.

Countries	Number of treatments from 2023 up to 2030		Treatment coverage by 2030 (%)		Number of HCV infections averted by 2030		Number of treatments per infection averted by 2030	
	Target 80% incidence reduction scenario^*^	Target incidence rate < 1 per 100,000 person-years scenario	Target 80% incidence reduction scenario^*^	Target incidence rate < 1 per 100,000 person-years scenario	Target 80% incidence reduction scenario^*^	Target incidence rate < 1 per 100,000 person-years scenario	Target 80% incidence reduction scenario^*^	Target incidence rate < 1 per 100,000 person-years scenario
Afghanistan	157,855 (93,171-272,909)	190,267 (114,559-317,363)	70.1 (67.2–73.1)	95.9 (92.7–97.9)	68,499 (34,815-134,797)	104,315 (52,244-206,369)	2.3 (2.0–2.7)	1.8 (1.5–2.2)
Algeria	32,360 (4,470-183,708)	41,004 (4,470-238,416)	57.0 (52.1–62.5)	79.4 (52.1–95.9)	11,038 (1,298-72,135)	15,834 (1,298-124,456)	2.9 (2.5–3.4)	2.6 (1.9–3.4)
Iran	79,408 (37,668-217,812)	108,520 (37,668-344,677)	46.9 (44.4–49.4)	68.3 (44.4–87.8)	9,180 (4,311-27,467)	13,587 (4,311-53,347)	8.7 (7.9–8.7)	8.0 (6.5–8.7)
Iraq	80,959 (41,542-266,293)	93,025 (41,542-287,141)	78.3 (77.2–85.1)	81.3 (77.2–97.0)	9,766 (5,916-22,740)	12,158 (5,258-41,903)	8.3 (7.0–11.7)	7.7 (6.9–7.9)
Jordan	16,762 (5,712-55,122)	16,828 (5,712-58,124)	78.4 (74.8–82.4)	79.0 (74.8–94.6)	2,578 (599–13,359)	2,598 (599–18,502)	6.5 (4.1–9.5)	6.5 (3.1–9.5)
Kuwait	17,639 (3,449-67,587)	35,241 (6,760-116,136)	40.2 (34.9–47.7)	95.4 (78.3–98.7)	3,115 (539–14,038)	8,732 (1,277-37,256)	5.7 (4.8–6.4)	4.0 (3.1–5.3)
Lebanon	2,610 (527–15,409)	3,470 (527–23,082)	45.7 (34.2–58.4)	62.5 (34.2–93.7)	401 (55–3,671)	559 (55–6,545)	6.5 (4.2–9.5)	6.2 (3.5–9.5)
Libya	53,079 (34,570-91,942)	66,828 (42,307-117,572)	68.8 (68.6–69.7)	93.1 (89.1–96.0)	3,939 (2,553-6,774)	6,006 (3,626-11,101)	13.5 (13.5–13.6)	11.1 (10.6–11.7)
Morocco	92,644 (49,590-168,718)	142,712 (78,226-251,166)	43.1 (41.9–44.9)	64.1 (63.2–66.2)	16,402 (8,311-31,637)	34,981 (18,295-64,799)	5.6 (5.3–6.0)	4.1 (3.9–4.3)
Oman	11,479 (5,762-22,810)	16,200 (8,276-31,152)	53.8 (51.2–56.5)	94.6 (89.8–97.2)	6,829 (3,279-14,222)	12,525 (5,877-26,131)	1.7 (1.6–1.8)	1.3 (1.2–1.4)
Palestine	13,111 (7,897-20,376)	13,111 (7,897-22,634)	71.0 (69.6–72.2)	71.0 (69.6–81.6)	757 (451–1,308)	757 (451–1,547)	17.3 (15.6–17.5)	17.3 (14.6–17.5)
Qatar	9,689 (9,599-11,423)	11,646 (11,569-13,784)	71.2 (71.1–72.1)	92.5 (92.5–93.9)	2,129 (2,086-2,566)	3,030 (3,002-3,707)	4.6 (4.5–4.6)	3.8 (3.7–3.9)
Saudi Arabia	75,308 (42,527-136,652)	140,986 (75,586-262,762)	40.9 (40.1–41.3)	87.5 (78.6–93.0)	9,137 (5,064-17,295)	21,952 (10,620-44,812)	8.2 (7.9–8.4)	6.4 (5.9–7.1)
Somalia	39,430 (10,838-117,635)	44,315 (10,838-129,873)	74.0 (67.4–80.6)	88.4 (67.4–96.7)	9,633 (1,807-38,473)	12,031 (1,807-51,683)	4.1 (3.1–6.0)	3.7 (2.5–6.0)
Sudan	180,416 (53,169-503,107)	211,912 (54,803-592,187)	73.3 (68.9–76.2)	91.2 (71.7–97.5)	28,047 (7,840-97,348)	38,359 (8,247-148,162)	6.4 (5.2–6.8)	5.5 (4.0–6.6)
Syria	54,479 (33,319-96,149)	54,479 (33,319-106,231)	71.1 (69.8–72.6)	71.1 (69.8–82.5)	3,129 (1,853-5,843)	3,129 (1,853-6,874)	17.4 (16.5–18.0)	17.4 (15.5–18.0)
Tunisia	24,477 (11,490-62,244)	24,553 (11,490-85,056)	55.5 (54.3–57.3)	55.5 (54.3–82.8)	943 (424–2,708)	947 (424–4,217)	25.9 (23.0–27.1)	25.9 (20.2–27.1)
United Arab Emirates	61,728 (31,934-126,268)	85,338 (44,907-164,004)	60.4 (58.8–63.7)	88.0 (87.9–88.9)	8,703 (4,262-20,227)	17,104 (8,184-37,118)	7.1 (6.2–7.5)	5.0 (4.4–5.5)
Yemen	119,749 (52,057-259,048)	208,382 (94,347-418,712)	45.7 (41.2–50.8)	93.6 (85.9–97.0)	24,688 (9,788-59,333)	57,532 (21,984-133,732)	4.9 (4.4–5.3)	3.6 (3.1–4.3)

^*^Incidence reduction was defined as the relative difference between incidence at a given time point and incidence in 2010. The year 2010 was chosen as a reference year for the target-80%-incidence-reduction scenario, as suggested earlier by the WHO (14, 44), and for consistency with the approach for Egypt and Pakistan (16, 17).

Table 4 shows the impact of the treatment program scenarios on HCV incidence rate in the total population of each of the 19 countries. HCV incidence rate, per 100,000 person-years in 2022, ranged between 3.5 in Tunisia and 61.1 in Oman, with a median of 16.1. In absence of the treatment program, incidence rate by 2030, per 100,000 person-years, ranged between 2.5 and 39.0 across countries with a median of 11.7.

**Table 4:** Impact of the treatment program scenarios on HCV incidence in 19 countries in the Middle East and North Africa.

Countries	Incidence rate in 2010 (per 100,000 person-years)	Incidence rate in 2022 (per 100,000 person-years)	Incidence rate by 2030 (per 100,000 person-years)			Incidence rate reduction strictly attributed to the treatment program scenarios by 2030 (%)	
			No-treatment intervention scenario	Target 80% incidence reduction scenario^*^	Target incidence rate < 1 per 100,000 person-years scenario	Target 80% incidence reduction scenario^*^	Target incidence rate < 1 per 100,000 person-years scenario
Afghanistan	74.9 (44.7–126.6)	49.6 (27.3–91.2)	37.8 (20.0–72.3)	8.3 (5.0–14.0)	1.0 (1.0–1.0)	78.0 (75.2–80.7)	97.4 (95.1–98.6)
Algeria	16.3 (2.5–81.8)	9.8 (1.4–55.3)	7.1 (0.9–42.8)	2.3 (0.4–11.6)	1.0 (0.4–1.0)	67.2 (61.0–72.8)	86.2 (61.0–97.7)
Iran	11.6 (5.9–31.4)	5.8 (2.9–16.4)	3.8 (1.9–11.0)	1.7 (0.9–4.7)	1.0 (0.9–1.0)	54.3 (52.4–56.9)	74.3 (52.4–91.1)
Iraq	13.5 (5.4–40.7)	8.4 (3.5–27.1)	6.2 (3.1–20.6)	1.1 (0.6–2.5)	1.0 (0.6–1.0)	82.3 (80.2–88.1)	84.1 (80.2–95.2)
Jordan	8.2 (2.4–30.9)	6.1 (1.6–27.1)	5.5 (1.4–27.4)	1.0 (0.3–3.7)	1.0 (0.3–1.0)	81.9 (78.1–86.4)	82.3 (78.1–96.2)
Kuwait	120.9 (25.7–412.5)	53.0 (10.6–197.8)	31.2 (6.1–123.2)	16.3 (3.5–54.4)	1.0 (1.0–1.0)	47.8 (42.4–55.9)	96.9 (83.8–99.2)
Lebanon	6.7 (1.6–33.9)	5.2 (1.1–32.2)	3.2 (0.6–23.4)	1.5 (0.4–7.6)	1.0 (0.4–1.0)	53.3 (40.2–67.6)	69.6 (40.2–95.8)
Libya	25.7 (16.6–44.9)	19.7 (12.7–34.2)	17.4 (11.3–30.3)	4.7 (3.0–8.1)	1.0 (1.0–1.0)	73.2 (73.1–73.2)	94.4 (91.3–96.8)
Morocco	45.5 (24.6–81.3)	23.6 (12.5–43.3)	14.7 (7.7–27.3)	6.9 (3.7–12.3)	1.0 (1.0–1.0)	53.1 (51.4–55.1)	93.2 (87.0–96.3)
Oman	107.1 (58.2–197.1)	61.1 (31.6–118.5)	39.0 (19.5–78.3)	12.3 (6.7–22.5)	1.0 (1.0–1.0)	68.5 (65.8–71.3)	97.5 (95.0–98.7)
Palestine	7.9 (5.1–13.1)	4.8 (3.1–8.2)	3.5 (2.2–6.1)	0.9 (0.6–1.5)	0.9 (0.6–1.0)	74.0 (72.8–74.9)	74.0 (72.8–83.9)
Qatar	36.9 (36.6–43.5)	22.5 (22.4–26.9)	19.7 (19.5–23.6)	4.4 (4.3–5.1)	1.0 (1.0–1.0)	77.9 (77.8–78.2)	95.0 (95.0–95.9)
Saudi Arabia	38.6 (22.0–69.8)	16.1 (9.1–29.4)	10.6 (5.9–19.5)	5.5 (3.2–9.9)	1.0 (1.0–1.0)	47.8 (46.9–49.1)	90.7 (83.5–95.0)
Somalia	21.6 (5.9–62.3)	15.5 (3.5–52.8)	11.7 (2.4–44.8)	2.3 (0.6–6.6)	1.0 (0.6–1.0)	80.3 (73.5–85.2)	91.6 (73.5–97.8)
Sudan	26.2 (9.0–79.8)	18.0 (5.6–58.5)	13.9 (4.1–47.2)	3.2 (1.1–9.6)	1.0 (1.0–1.0)	77.2 (73.3–79.7)	92.9 (75.9–97.9)
Syria	6.3 (4.0–10.7)	4.5 (2.7–7.9)	3.6 (2.1–6.5)	0.9 (0.6–1.5)	0.9 (0.6–1.0)	74.4 (73.2–76.2)	74.4 (73.2–84.9)
Tunisia	6.3 (2.9–17.0)	3.5 (1.6–9.7)	2.5 (1.1–7.0)	1.0 (0.5–2.6)	1.0 (0.5–1.0)	60.8 (60.1–62.6)	61.0 (60.1–86.1)
United Arab Emirates	76.7 (39.8–155.9)	39.0 (19.8–83.7)	27.0 (13.5–60.3)	8.7 (4.5–17.3)	1.0 (1.0–1.0)	67.9 (66.3–71.2)	96.3 (92.7–98.3)
Yemen	95.5 (45.3–187.1)	42.9 (19.2–90.2)	22.9 (9.9–50.3)	10.4 (5.0–20.3)	1.0 (1.0–1.0)	54.3 (49.6–59.6)	95.7 (90.1–98.1)

^*^Incidence reduction was defined as the relative difference between incidence at a given time point and incidence in 2010. The year 2010 was chosen as a reference year for the target-80%-incidence-reduction scenario, as suggested earlier by the WHO (14, 44), and for consistency with the approach for Egypt and Pakistan (16, 17).

In presence of the treatment program, incidence rate by 2030, per 100,000 person-years, ranged between 0.9 and 16.3 with a median of 3.2 in the target-80%-incidence-reduction scenario. Palestine and Syria reached the goal of incidence rate < 1 per 100,000 person-years within this scenario. All countries reached this goal in the target incidence rate < 1 per 100,000 person-years scenario, as expected. Incidence rate reduction by 2030 strictly attributed to the treatment program ranged between 47.8 and 81.9% with a median of 68.5% in the target-80%-incidence-reduction scenario, and between 61.0 and 97.5% with a median of 90.7% in the target incidence rate < 1 per 100,000 person-years scenario.

**Figure 1:**
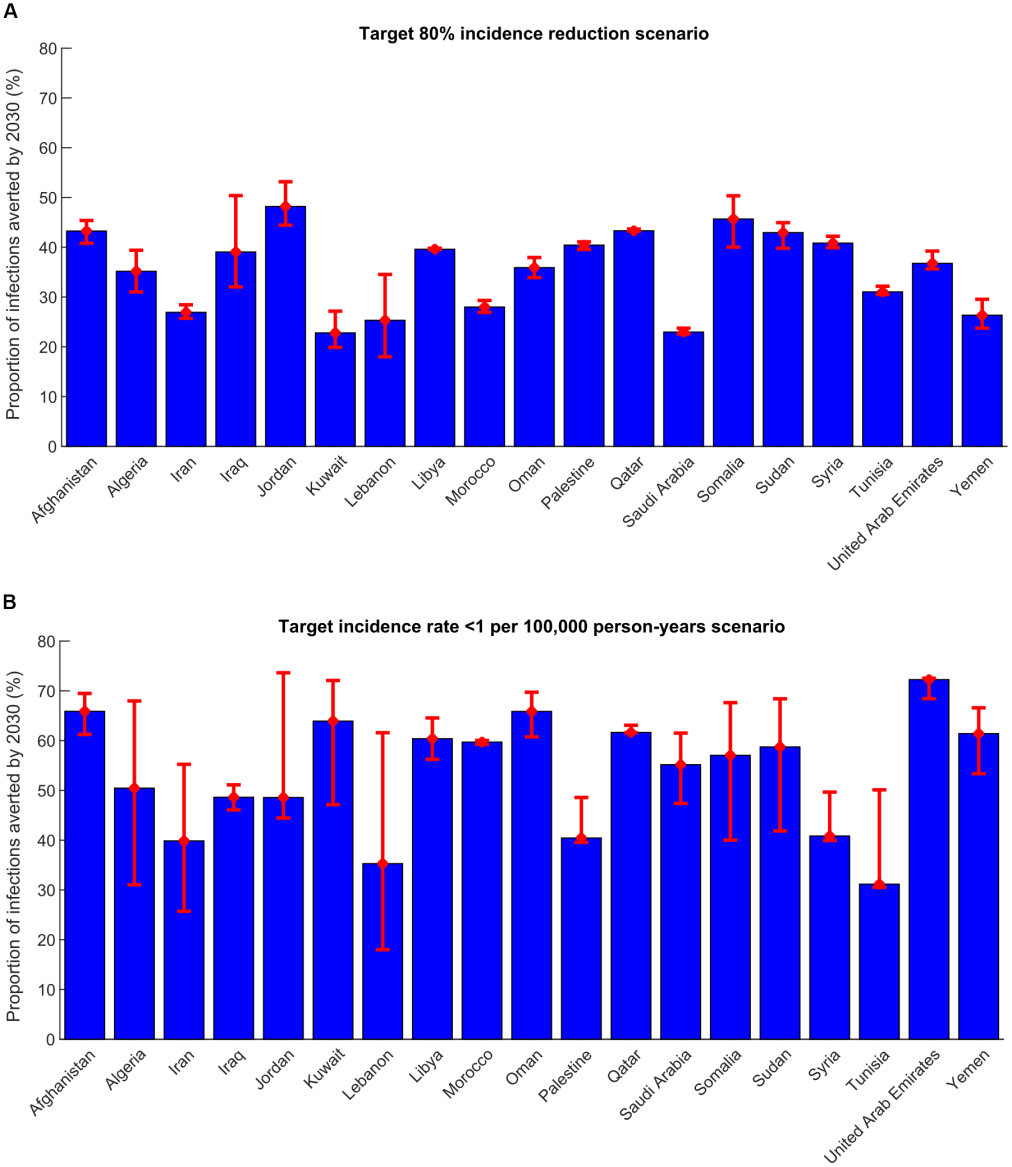
Proportion of infections averted by 2030 in 19 countries in the Middle East and North Africa in the **(A)** target-80%-incidence-reduction scenario and **(B)** target incidence rate  < 1 per 100,000 person-years scenario. This proportion is defined as the total number of infections averted by the treatment program between 2023 and 2030 divided by the total number of new infections in the no-intervention counter-factual scenario within the same timeframe.

Other program indicators are shown in Table 3 and Figure 1. The number of treatments administrated between 2023 and 2030 varied widely by country depending on the country’s population size and number of chronically infected individuals in need of treatment. Treatment coverage varied also by country depending on the current prevalence of chronic infection and incidence. The number of treatments required to prevent one new infection varied from 1.3 to 25.9 across countries. Despite the significant disparities in the number of chronically infected individuals requiring treatment among the 19 MENA countries, the range of treatments needed to prevent one new infection remained relatively narrow.

In the target-80%-incidence-reduction scenario, the number of treatments administrated between 2023 and 2030 ranged between 2,610 in Lebanon and 180,416 in Sudan, with a median of 53,079. Treatment coverage in 2030 ranged between 40.2% in Kuwait and 78.4% in Jordan, with a median of 60.4%. Number of infections averted by 2030 ranged from 401 in Lebanon and 68,499 in Afghanistan, with a median of 8,703. The proportion of infections averted by 2030 out of all incident infections was lowest in Kuwait and highest in Jordan (Figure 1). Number of treatments required to avert one new infection by 2030 ranged between 1.7 in Oman and 25.9 in Tunisia, with a median of 6.5 (Table 3).

In the target incidence rate < 1 per 100,000 person-years scenario, the number of treatments administrated between 2023 and 2030 ranged between 3,470 in Lebanon and 211,912 in Sudan, with a median of 54,479 (Table 3). Treatment coverage in 2030 ranged between 55.5% in Tunisia and 95.9% in Afghanistan, with a median of 87.5%. Number of infections averted by 2030 ranged between 559 in Lebanon and 104,315 in Afghanistan, with a median of 12,158. The proportion of infections averted by 2030 out of all incident infections was lowest in Tunisia and highest in United Arab Emirates (Figure 1). Number of treatments required to avert one new infection by 2030 ranged between 1.3 in Oman and 25.9 in Tunisia, with a median of 5.5 (Table 3).

Supplementary Tables S2–S4 show the results of the scenario of targeting an incidence of ≤5 per 100,000 people per year by 2030. This scenario was not applicable in several countries, including Iran, Lebanon, Palestine, Syria, and Tunisia, as they were already on track or projected to achieve an incidence of ≤5 per 100,000 people per year by 2030 without requiring treatment scale-up (Supplementary Table S3). However, the overall results in this scenario still highlight the benefits of the treatment program and align with the findings from the two primary scenarios.

Median treatment coverage for 2030 varied across target scenarios: 49.1% in the target incidence ≤5 per 100,000 people per year scenario, 60.4% in the target-80%-incidence-reduction scenario, and 87.5% in the target incidence rate < 1 per 100,000 person-years scenario. The median number of infections averted by 2030 also varied: 4,280 in the target incidence ≤5 per 100,000 people per year scenario, 8,703 in the target-80%-incidence-reduction scenario, and 12,158 in the target incidence rate < 1 per 100,000 person-years scenario. The median number of treatments required to prevent one new infection by 2030 ranged from 6.9 in the target incidence ≤5 per 100,000 people per year scenario to 6.5 in the target-80%-incidence-reduction scenario, and 5.5 in the target incidence rate < 1 per 100,000 person-years scenario.

**Figure 2:**
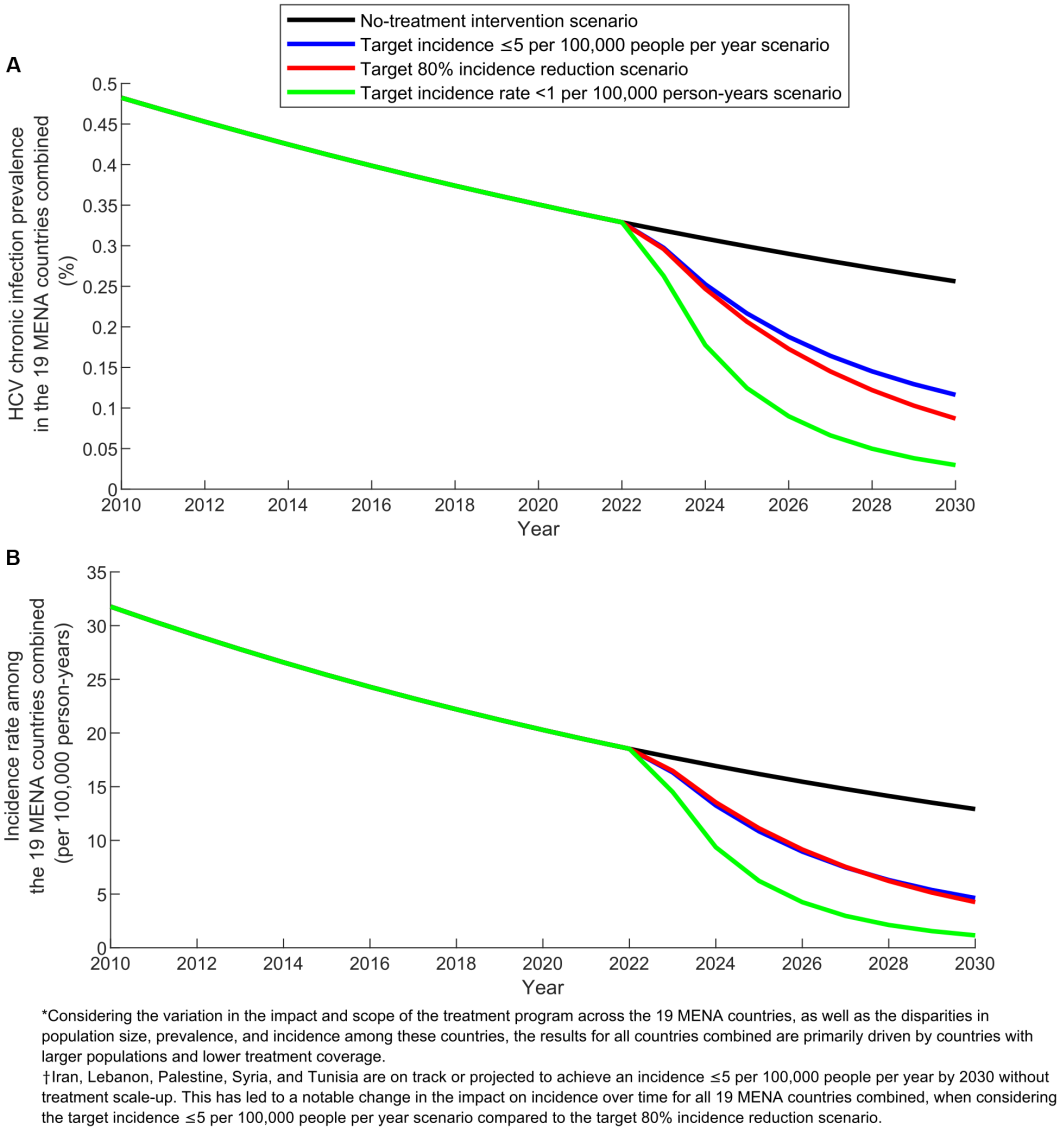
Projected outcomes of the treatment program scenarios in the combined 19 countries of the Middle East and North Africa (MENA). **(A)** Prevalence of HCV chronic infection and **(B)** Incidence rate in the baseline scenario without treatment intervention compared to the scenarios with target incidence ≤5 per 100,000 people per year, target 80% incidence reduction, and target incidence rate  < 1 per 100,000 person-years.

Figure 2 illustrates the predicted impact of the three program scenarios over time in the combined 19 MENA countries. The prevalence of chronic HCV infection, estimated at 0.3% in 2022 (prior to program initiation), was projected to decline to 0.0–0.1% by 2030 under the different treatment scenarios, compared to 0.2% in the no-treatment intervention scenario. The HCV incidence rate per 100,000 person-years, which was at 18.5 in 2022, was projected to decrease to 1.0–4.6 by 2030 across the various treatment scenarios, compared to 13.0 in the no-treatment intervention scenario.

**Figure 3:**
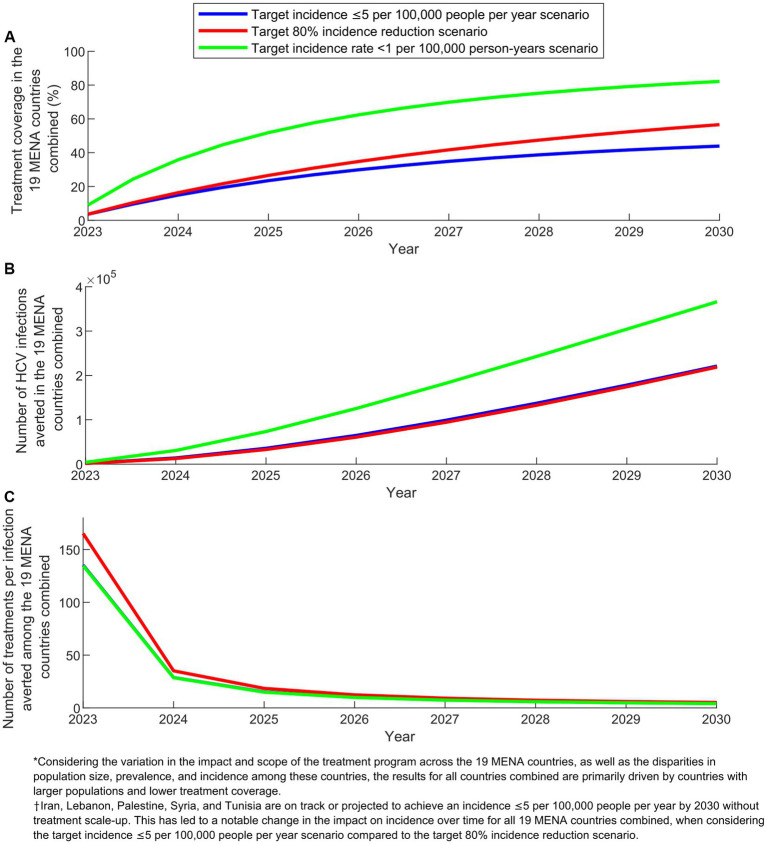
Key program indicators for the treatment program scenarios in the combined 19 countries of the Middle East and North Africa (MENA). **(A)** Treatment coverage, **(B)** Number of HCV infections averted, and **(C)** Number of treatments per infection averted in the scenarios with target incidence ≤5 per 100,000 people per year, target 80% incidence reduction, and target incidence rate  < 1 per 100,000 person-years.

Figure 3 displays key program indicators over time for the three program scenarios in the combined 19 MENA countries. In 2030, treatment coverage ranged from 44.0% in the target incidence ≤5 per 100,000 people per year scenario to 82.0% in the target incidence rate < 1 per 100,000 person-years scenario. The number of infections averted by 2030 varied between 218,914 in the target 80% incidence reduction scenario and 366,136 in the target incidence rate < 1 per 100,000 person-years scenario. The number of treatments required to prevent one new infection by 2030 remained around 4.5 across the scenarios.

In comparing the impact over time of the different scenarios at the combined level of the 19 countries, an important caveat needs to be noted when explaining the observed results. The scenario targeting an incidence of ≤5 per 100,000 people per year does not apply to Iran, Lebanon, Palestine, Syria, and Tunisia. This is because these countries are already on track or projected to achieve an incidence of ≤5 per 100,000 people per year by 2030 (Supplementary Table S3). This caveat accounts for the varying relative impact between the scenario aiming for an incidence of ≤5 per 100,000 people per year and the scenario targeting an 80% reduction in incidence (Figures 2, 3).

## Discussion

Current prevalence of chronic infection is ≤1% in the 19 MENA countries investigated in this study, substantially lower than that in Egypt and Pakistan, the two MENA countries most affected by HCV infection (16, 17). Incidence of HCV infection appears to be on a trajectory of decline, confirming other analyses reporting such declines (24, 25). However, this decline is not fast enough to achieve the elimination target by 2030.

The impact of HCV-TasP in the 19 MENA countries is relatively lower compared to the estimated impact in Egypt and Pakistan (16, 17). This is primarily attributed to the significantly lower prevalence of chronic HCV infection in those 19 countries. As a result, the number of infections averted in Egypt and Pakistan exceeds the total number of averted infections in the combined 19 MENA countries.

However, HCV-TasP was shown to provide still a strategic approach to control the epidemic and to reach the elimination target by 2030 in these 19 countries. While HCV treatment is indicated at the individual level to prevent disease sequelae such as fibrosis, cirrhosis, and liver cancer (4, 5), the impact of the treatment on reducing (indirectly) the onward transmission of the infection is substantial and could lead to elimination if carriers of this infection are identified and treated.

Most of the of the projected incidence declines following roll-out of HCV-TasP, in the range of 61.0–97.5% across countries in the target incidence rate < 1 per 100,000 person-years scenario, are attributed to the treatment program and not to natural evolution of the epidemic. The impact of the treatment program materialized rapidly within only few years of operation. The number of DAA treatments needed to avert one infection was relatively low with a median of 5.5, even though this group of MENA countries had concentrated epidemics with a low chronic infection prevalence.

These findings confirm the high public health value of the HCV-TasP approach, as an effective prevention intervention against transmission, just as it is a cost-effective and cost-saving treatment intervention against disease sequelae. The availability of DAA generics in multiple countries at a cost <$100 per drug regimen highlights the historical opportunity of eliminating this infection at a relatively low program cost, as demonstrated recently for Egypt (47), one of the MENA countries. This cost is currently minimal compared to a few years ago. This is significant considering the potential future healthcare costs that are being averted, not to mention other costs in terms of productivity, disability, and premature mortality.

While this historical opportunity of elimination is accessible, it hinges on overcoming a major hurdle, that of identifying chronically infected individuals. This challenge is especially acute in this group of 19 countries because of the low prevalence of chronic infection. Most carriers of this virus are unaware of their infection status and typically may not be diagnosed before onset of serious HCV disease sequelae. Mass HCV screening in the population, such as in the generalized epidemic of Egypt (47), is not likely to be justified nor cost-effective with the low prevalence of chronic infection in these countries. Obtaining current and reliable data on the number of people diagnosed and treated for HCV in each country, including both the public and private healthcare sectors, is generally challenging. This lack of data hampers the assessment of progress in scaling up treatment efforts.

The most feasible testing strategy to optimize the yield of testing and program efficiency is perhaps that of targeted testing for specific subpopulations (23). Risk of exposure varies immensely by subpopulation and shows a distinctive hierarchy, particularly in concentrated epidemics such as those present in these 19 MENA countries (23). Efficiency of testing strategies can be enhanced by population prioritization by risk of exposure as demonstrated previously (23). In concentrated MENA epidemics, the number of tests needed to identify one chronic infection was 2.8 for people who inject drugs, 8.6 for populations with liver conditions, 5.1 for populations with high-risk healthcare exposures, but was much higher at 222.2 for general populations (23).

Though this group of countries had all low prevalence of chronic infection, there were differences in the impact of the HCV-TasP program. The level of incidence rate and its rate of decline due to natural evolution of the epidemic varied considerably across countries and this caused variation in HCV-TasP impact. Some countries had slowly declining incidence rate while others had rapidly declining incidence rate. The relationship between chronic infection prevalence and incidence rate also varied across countries, leading to variation in HCV-TasP impact. In some countries, current chronic infection prevalence was high relative to current incidence rate, as the majority of chronic infections reflected historical exposures due to a past cohort effect (48); that is prevalence did not reflect recent exposures or current incidence rate. Demographic factors also played a role. Age distribution of the population varied across countries and several countries were affected by high rates of immigration, such as Kuwait, Oman, Qatar, and United Arab Emirates, where a large proportion of the population are young/mid-age expatriates from other regions. Impact of the elimination scenario versus the target-80%-incidence-reduction scenario differed across countries for these same factors as well as the different rate of scale-up of the treatment in each of these scenarios.

While the impact of HCV-TasP varied across individual countries, the combined results for the 19 MENA countries demonstrated significant regional-level benefits. All program scenarios showed substantial reductions in both incidence and chronic infection prevalence. In the target incidence rate < 1 per 100,000 person-years scenario, where a treatment coverage of over 80% was achieved by 2030, approximately 1.5 million individuals received treatment, leading to the prevention of nearly 370,000 infections. By 2030, every 4.5 treatments prevented one infection. These findings highlight the substantial impact and effectiveness of the treatment program at a regional level.

This study has limitations. Number of Ab prevalence measures varied across countries and for some countries the number of measures over time was not adequate to capture concrete time trends in Ab prevalence. Available Ab prevalence measures may not also be representative of actual prevalence with the small number of seroprevalence surveys done in the considered countries. We assumed any chronically infected individual can be treated, but treatment may not be indicated for all. We assumed that coverage of treatment has been negligible so far in these MENA countries, but this may not apply to all countries. Some of the biological parameters used in the model were based on the existing literature including studies on people who inject drugs, which may affect their generalizability to the general population. Future incidence can be uncertain as it can depend on unpredictable factors, such as scale-up of other coincidental interventions. We used a complex mathematical model to describe HCV transmission in the population, but predictions may be contingent on type of model and its input data.

To partially address these limitations, multivariable uncertainty analyses were conducted to estimate the level of uncertainty surrounding the model projections. These analyses revealed that the extent of uncertainty varied across countries, underscoring the importance of obtaining improved input data on Ab prevalence in specific countries. Enhanced data would allow the model to generate more precise projections. Conducting multiple rounds of population-based national surveys, similar to the ones conducted in Egypt (49, 50), would have significantly enhanced the model’s capability to capture the levels and trends of the epidemic in the 19 MENA countries.

If new national or regional surveys are carried out in these countries in the near future, the data from these surveys can be incorporated into the modeling approach to generate updated results that account for the newly available evidence. Therefore, while the study results offer estimates for initiating treatment programs, planning activities, and allocating resources, they represent an ongoing effort that requires regular updates as more input data become available. These updates will contribute to improving the certainty and reliability of the estimates.

In conclusion, this study investigated the impact of the HCV-TasP program in 19 MENA countries using different program scenarios. Among these scenarios, the one targeting an incidence rate of less than 1 per 100,000 person-years demonstrated the most significant impact. However, implementing this scenario may pose challenges for certain countries due to issues such as screening limitations, accessibility to DAAs, costs, and adaptability of the public health infrastructure. Each country should determine a target scenario that suits their specific circumstances. This study presented three different scenarios as options for consideration. Regardless of the scenario chosen, the projected outcomes consistently demonstrated that HCV-TasP is an effective and indispensable intervention for controlling the epidemic and achieving the goal of HCV elimination by 2030. These 19 MENA countries have an opportunity to avert many new infections and eliminate HCV and much of its disease sequelae by 2030, by adopting the HCV-TasP approach.

## Data availability statement

The original contributions presented in the study are included in the article/Supplementary material, further inquiries can be directed to the corresponding authors.

## Author contributions

HHA designed the mathematical model, conducted the analyses, and wrote the first draft of the article. SM and HC supported the model parameterization and conducted statistical analyses. LJA conceived and led the design of the study and model, analyses, and drafting of the article. All authors contributed to the article and approved the submitted version.

## Funding

This publication was made possible by NPRP grant number 12S-0216-190094 from the Qatar National Research Fund (a member of Qatar Foundation; https://www.qnrf.org). HHA acknowledges the support of Qatar University QUCG-CAS-23/24–114. The statements made herein are solely the responsibility of the authors. The funders had no role in study design, data collection and analysis, decision to publish, or preparation of the manuscript. The authors alone are responsible for the views expressed in this publication and they do not necessarily represent the views, decisions, or policies of World Health Organization. The authors are also grateful for infrastructure support provided by the Biostatistics, Epidemiology, and Biomathematics Research Core at Weill Cornell Medicine-Qatar.

## Conflict of interest

The authors declare that the research was conducted in the absence of any commercial or financial relationships that could be construed as a potential conflict of interest.

## Publisher’s note

All claims expressed in this article are solely those of the authors and do not necessarily represent those of their affiliated organizations, or those of the publisher, the editors and the reviewers. Any product that may be evaluated in this article, or claim that may be made by its manufacturer, is not guaranteed or endorsed by the publisher.

## References

[1] Stanaway JD Flaxman AD Naghavi M Fitzmaurice C Vos T Abubakar I. The global burden of viral hepatitis from 1990 to 2013: findings from the global burden of disease study 2013. Lancet. (2016) 388:1081–8. doi: 10.1016/S0140-6736(16)30579-7, PMID: 27394647 PMC5100695

[2] World Health Organization (2014) Hepatitis C: Fact sheet Geneva, Switzerland (updated April 2014). Available at: http://www.who.int/mediacentre/factsheets/fs164/en/.

[3] Centers for Disease Control and Prevention (2013). Hepatitis C Atlanta, USA updated August 1, 2013. Available at: http://wwwnc.cdc.gov/travel/yellowbook/2014/chapter-3-infectious-diseases-related-to-travel/hepatitis-c.

[4] Shepard CW Finelli L Alter MJ. Global epidemiology of hepatitis C virus infection. Lancet Infect Dis. (2005) 5:558–67. doi: 10.1016/S1473-3099(05)70216-4 16122679

[5] Maheshwari A Ray S Thuluvath PJ. Acute hepatitis C. Lancet. (2008) 372:321–32. doi: 10.1016/S0140-6736(08)61116-2 18657711

[6] World Health Organization (WHO). Global hepatitis report, (2017). Available at: http://www.who.int/hepatitis/publications/global-hepatitis-report2017/en/ (Accessed 7 October 2021).

[7] World Health Organization. Epidemiology of hepatitis C virus in the WHO eastern Mediterranean region: Implications for strategic action. Cairo: WHO Regional Office for the Eastern Mediterranean (2020).

[8] World Health Organization. Global progress report on HIV, viral hepatitis and sexually transmitted infections, 2021: Accountability for the global health sector strategies 2016–2021: Actions for impact: Web annex 2: Data methods (2021).

[9] Global Burden of Disease Collaborative Network. Global burden of disease study 2019 (GBD 2019) results. Seattle, United States: Institute for Health Metrics and Evaluation (IHME) (2020).

[10] Flamm SL. Advances in the treatment of hepatitis C virus infection from EASL 2015. Gastroenterol Hepatol. (2015) 11:1–23. Available at: https://pubmed.ncbi.nlm.nih.gov/26504459/

[11] Manns MP Buti M Gane E Pawlotsky J-M Razavi H Terrault N. Hepatitis C virus infection. Nat Rev Dis Primers. (2017) 3:1–19. doi: 10.1038/nrdp.2017.6 28252637

[12] Vermehren J Park JS Jacobson IM Zeuzem S. Challenges and perspectives of direct antivirals for the treatment of hepatitis C virus infection. J Hepatol. (2018) 69:1178–87. doi: 10.1016/j.jhep.2018.07.002, PMID: 30006068

[13] World Health Organization. Combating hepatitis B and C to reach elimination by 2030. Geneva, Switzerland: World Health Organization (2016).

[14] World Health Organization. Global Health sector strategy on viral hepatitis, 2016–2021. Geneva, Switzerland: World Health Organization (2015).

[15] World Health Organization. Global health sector strategies on, respectively, HIV, viral hepatitis and sexually transmitted infections for the period 2022–2030. Geneva, Switzerland: World Health Organization (2022).

[16] Ayoub HH Abu-Raddad LJ. Impact of treatment on hepatitis C virus transmission and incidence in Egypt: a case for treatment as prevention. J Viral Hepat. (2017) 24:486–95. doi: 10.1111/jvh.12671, PMID: 28039923

[17] Ayoub HH Abu-Raddad LJ. Treatment as prevention for hepatitis C virus in Pakistan: mathematical modelling projections. BMJ Open. (2019) 9:e026600. doi: 10.1136/bmjopen-2018-026600, PMID: 31133586 PMC6537971

[18] Granich RM Gilks CF Dye C De Cock KM Williams BG. Universal voluntary HIV testing with immediate antiretroviral therapy as a strategy for elimination of HIV transmission: a mathematical model. Lancet. (2009) 373:48–57. doi: 10.1016/S0140-6736(08)61697-9 19038438

[19] Ayoub HH Al Kanaani Z Abu-Raddad LJ. Characterizing the temporal evolution of the hepatitis C virus epidemic in Pakistan. J Viral Hepat. (2018) 25:670–9. doi: 10.1111/jvh.12864, PMID: 29345847

[20] Delva W Wilson DP Abu-Raddad L Gorgens M Wilson D Hallett TB. HIV treatment as prevention: principles of good HIV epidemiology modelling for public health decision-making in all modes of prevention and evaluation. PLoS Med. (2012) 9:e1001239. doi: 10.1371/journal.pmed.1001239 22802729 PMC3393657

[21] Grebely J Page K Sacks-Davis R Loeff MS Rice TM Bruneau J. The effects of female sex, viral genotype, and IL28B genotype on spontaneous clearance of acute hepatitis C virus infection. Hepatology. (2014) 59:109–20. doi: 10.1002/hep.26639, PMID: 23908124 PMC3972017

[22] Harfouche M Chemaitelly H Kouyoumjian SP Mahmud S Chaabna K Al-Kanaani Z. Hepatitis C virus viremic rate in the Middle East and North Africa: systematic synthesis, meta-analyses, and meta-regressions. PLoS One. (2017) 12:e0187177. doi: 10.1371/journal.pone.0187177, PMID: 29088252 PMC5663443

[23] Chemaitelly H Mahmud S Kouyoumjian SP Al-Kanaani Z Hermez JG Abu-Raddad LJ. Who to test for hepatitis C virus in the Middle East and North Africa?: pooled analyses of 2,500 prevalence measures, including 49 million tests. Hepatol Commun. (2019) 3:325–39. doi: 10.1002/hep4.1310, PMID: 30859146 PMC6396361

[24] Mahmud S Chemaitelly H Alaama AS Hermez JG Abu-Raddad LJ. Characterizing trends and associations for hepatitis C virus antibody prevalence in the Middle East and North Africa: meta-regression analyses. Sci Rep. (2022) 12:20637. doi: 10.1038/s41598-022-25086-5, PMID: 36450850 PMC9712517

[25] Mahmud S Chemaitelly H Alaama AS Hermez JG Abu-Raddad L. Hepatitis C virus among blood donors and general population in Middle East and North Africa: Meta-analyses and meta-regressions. World J. Meta-Analysis. (2022) 10:12–24. doi: 10.13105/wjma.v10.i1.12

[26] Mahmud S Mumtaz GR Chemaitelly H Al Kanaani Z Kouyoumjian SP Hermez JG. The status of hepatitis C virus infection among people who inject drugs in the Middle East and North Africa. Addiction. (2020) 115:1244–62. doi: 10.1111/add.14944, PMID: 32009283 PMC7318323

[27] Garnett GP Anderson RM. Factors controlling the spread of HIV in heterosexual communities in developing countries: patterns of mixing between different age and sexual activity classes. Philos Trans R Soc Lond B Biol Sci. (1993) 342:137–59. doi: 10.1098/rstb.1993.0143 7904355

[28] Awad SF Abu-Raddad LJ. Could there have been substantial declines in sexual risk behavior across sub-Saharan Africa in the mid-1990s? Epidemics. (2014) 8:9–17. doi: 10.1016/j.epidem.2014.06.001, PMID: 25240899

[29] United Nations Department of Economic and Social Affairs. World population prospects, the 2022 revision (2022).

[30] Chemaitelly H Mahmud S Rahmani AM Abu-Raddad LJ. The epidemiology of hepatitis C virus in Afghanistan: systematic review and meta-analysis. Int J Infect Dis. (2015) 40:54–63. doi: 10.1016/j.ijid.2015.09.011 26417880

[31] Kouyoumjian S Chemaitelly H Abu-Raddad LJ. Characterizing hepatitis C virus epidemiology in Egypt: systematic reviews, meta-analyses, and meta-regressions. Sci Rep. (2018) 8:1661. doi: 10.1038/s41598-017-17936-4, PMID: 29374178 PMC5785953

[32] Mohamoud YA Mumtaz GR Riome S Miller D Abu-Raddad LJ. The epidemiology of hepatitis C virus in Egypt: a systematic review and data synthesis. BMC Infect Dis. (2013) 13:288. doi: 10.1186/1471-2334-13-288 23799878 PMC3702438

[33] Mohamoud YA Riome S Abu-Raddad LJ. Epidemiology of hepatitis C virus in the Arabian gulf countries: systematic review and meta-analysis of prevalence. Int J Infect Dis. (2016) 46:116–25. doi: 10.1016/j.ijid.2016.03.012 26996460

[34] Mahmud S Akbarzadeh V Abu-Raddad LJ. The epidemiology of hepatitis C virus in Iran: systematic review and meta-analyses. Sci Rep. (2018) 8:150. doi: 10.1038/s41598-017-18296-9, PMID: 29317673 PMC5760657

[35] Fadlalla FA Mohamoud YA Mumtaz GR Abu-Raddad LJ. The epidemiology of hepatitis C virus in the Maghreb region: systematic review and meta-analyses. PLoS One. (2015) 10:e0121873. doi: 10.1371/journal.pone.0121873, PMID: 25803848 PMC4372394

[36] Al Kanaani Z Mahmud S Kouyoumjian SP Abu-Raddad LJ. The epidemiology of hepatitis C virus in Pakistan: systematic review and meta-analyses. R Soc Open Sci. (2018) 5:180257. doi: 10.1098/rsos.180257, PMID: 29765698 PMC5936963

[37] Chemaitelly H Chaabna K Abu-Raddad LJ. The epidemiology of hepatitis C virus in the Fertile Crescent: systematic review and Meta-analysis. PLoS One. (2015) 10:e0135281. doi: 10.1371/journal.pone.0135281, PMID: 26296200 PMC4546629

[38] Chaabna K Kouyoumjian SP Abu-Raddad LJ. Hepatitis C virus epidemiology in Djibouti, Somalia, Sudan, and Yemen: systematic review and Meta-analysis. PLoS One. (2016) 11:e0149966. doi: 10.1371/journal.pone.0149966, PMID: 26900839 PMC4764686

[39] Mahmud S Al Kanaani Z Abu-Raddad LJ. Characterization of the hepatitis C virus epidemic in Pakistan. BMC Infect Dis. (2019) 19:809. doi: 10.1186/s12879-019-4403-7, PMID: 31521121 PMC6744714

[40] MATLAB®. The language of technical computing. Massachusetts: The MathWorks, Inc (2015).

[41] Lagarias JC Reeds JA Wright MH Wright PE. Convergence properties of the Nelder--Mead simplex method in low dimensions. SIAM J Optim. (1998) 9:112–47. doi: 10.1137/S1052623496303470

[42] Woods RD Saxon DS. Diffuse surface optical model for nucleon-nuclei scattering. Phys Rev. (1954) 95:577–8. doi: 10.1103/PhysRev.95.577

[43] Velicia FF. On the moments of a (WS) β distribution. J Phys A Math Gen. (1987) 20:2293–306. doi: 10.1088/0305-4470/20/9/017

[44] Hirnschall G. WHO 2016–2021 Global Health sector strategy viral hepatitis. The first meeting of the National Focal Points for viral hepatitis. Cairo, Egypt (2015).

[45] Estes C Abdel-Kareem M Abdel-Razek W Abdel-Sameea E Abuzeid M Gomaa A. Economic burden of hepatitis C in Egypt: the future impact of highly effective therapies. Aliment Pharmacol Ther. (2015) 42:696–706. doi: 10.1111/apt.13316, PMID: 26202593 PMC5034818

[46] Lönnroth K Migliori GB Abubakar I D'Ambrosio L De Vries G Diel R. Towards tuberculosis elimination: An action framework for low-incidence countries. Switzerland: World Health Organization (2015). 10.1183/09031936.00214014 PMC4391660 25792630

[47] Waked I Esmat G Elsharkawy A El-Serafy M Abdel-Razek W Ghalab R. Screening and treatment program to eliminate hepatitis C in Egypt. N Engl J Med. (2020) 382:1166–74. doi: 10.1056/NEJMsr1912628, PMID: 32187475

[48] Ayoub HH Chemaitelly H Kouyoumjian SP Abu-Raddad LJ. Characterizing the historical role of parenteral antischistosomal therapy in hepatitis C virus transmission in Egypt. Int J Epidemiol. (2020) 49:798–809. doi: 10.1093/ije/dyaa052, PMID: 32357208 PMC7394952

[49] El-Zanaty F Way A. Egypt demographic and health survey 2008. Cairo, Egypt: Ministry of Health, El-Zanaty and Associates, and Macro International (2009).

[50] Ministry of Health and Population (Egypt). El-Zanaty and associates ICF international. Egypt health issues survey 2015. Cairo, Egypt and Rockville, Maryland, USA (2015).

