# Supplementary material for: Treatment as prevention for hepatitis C virus in the Middle East and North Africa: a modeling study
Source: Front Public Health. 2023 Jul 13;11:1187786. doi: 10.3389/fpubh.2023.1187786 (PMC10374017; doi:10.3389/fpubh.2023.1187786)
Supplement: Supplementary file 1 [file Data_Sheet_1.docx]

Supplementary Material

Treatment as prevention for hepatitis C virus in the Middle East and North Africa: A modeling study

**Houssein H. Ayoub^1^, Sarwat Mahmud^2,3^, Hiam Chemaitelly^2,3,4^, and Laith J. Abu-Raddad^2,3,4,5.6^**

^1^Mathematics Program, Department of Mathematics, Statistics, and Physics, College of Arts and Sciences, Qatar University, Doha, Qatar

^2^Infectious Disease Epidemiology Group, Weill Cornell Medicine-Qatar, Cornell University, Doha, Qatar

^3^World Health Organization Collaborating Centre for Disease Epidemiology Analytics on HIV/AIDS, Sexually Transmitted Infections, and Viral Hepatitis, Weill Cornell Medicine–Qatar, Cornell University, Qatar Foundation – Education City, Doha, Qatar

^4^Department of Population Health Sciences, Weill Cornell Medicine, Cornell University, New York, New York, USA

^5^Department of Public Health, College of Health Sciences, QU Health, Qatar University, Doha, Qatar

^6^College of Health and Life Sciences, Hamad bin Khalifa University, Doha, Qatar

*** Correspondence:**Dr. Houssein H. Ayoub, Mathematics Program, Department of Mathematics, Statistics, and Physics, College of Arts and Sciences, Qatar University, P.O. Box 2713, Doha, Qatar. Telephone: +(974) 4403-7543. E-mail: [hayoub@qu.edu.qa](mailto:hayoub@qu.edu.qa).

Professor Laith J. Abu-Raddad, Infectious Disease Epidemiology Group, World Health Organization Collaborating Centre for Disease Epidemiology Analytics on HIV/AIDS, Sexually Transmitted Infections, and Viral Hepatitis, Weill Cornell Medicine - Qatar, Qatar Foundation - Education City, P.O. Box 24144, Doha, Qatar. Telephone: +(974) 4492-8321. Fax: +(974) 4492-8333. E-mail: [lja2002@qatar-med.cornell.edu](mailto:lja2002@qatar-med.cornell.edu)

**Figure S1.** Schematic diagram illustrating the basic structure of the deterministic mathematical model used to describe HCV transmission dynamics in the population. The detailed structure of this model and its description are found in References (1-3). In this figure, solid lines denote progression from one population compartment to the next, while dashed lines denote backward movement from the present population compartment to the previous one.


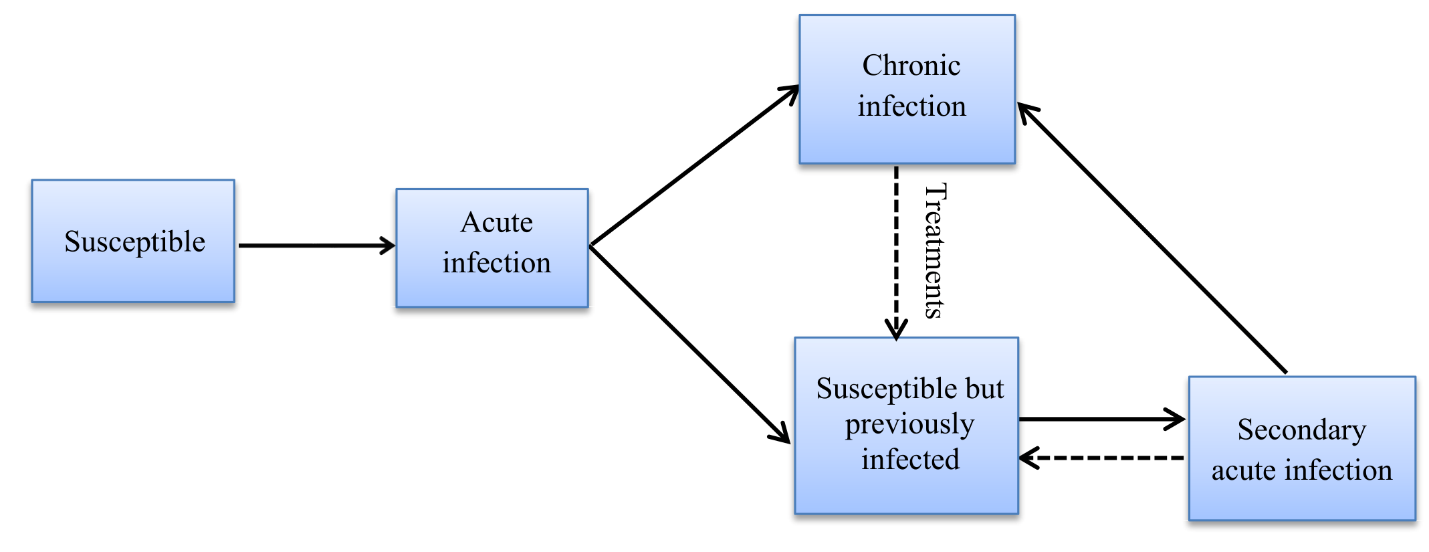


**Table S1. Model assumptions in terms of parameter values.**

| **Parameter** | **Value** | **Justification** | **Sources** |
| --- | --- | --- | --- |
| Transmission probability per contact in chronic infection stage | 0.048 | Combination of empirical data and quantitative estimates | (4-9) |
| Transmission probability per contact in primary acute infection | 0.1296 | Based on the relative level of viral load with respect to chronic infection stage | (6) |
| Transmission probability per contact in secondary acute infection | 0.0648 | Reduction of 50% in viral load during secondary acute infection with respect to initial primary acute infection | (10) |
| Duration of primary acute HCV infection stage | 16.5 weeks | Direct measurement from a prospective cohort study | (11) |
| Duration of secondary acute HCV infection stage | 4.125 weeks | Direct measurement from prospective cohort studies | (10, 12) |
| Fraction of individuals who clear their primary acute HCV infection spontaneously (first infection) | 25% | Direct measurement from a prospective cohort study | (11) |
| Fraction of individuals who clear their secondary acute HCV infection spontaneously (reinfection) | 83% | Direct measurement from a prospective cohort study | (10) |
| Duration that an individual spends in a specific $i$ risk group | 12 - 40 years | A hierarchy of durations based on risk groups starting with an injecting career of 12 years among people who inject drugs (highest risk group) up to a low risk duration of 40 years (lowest risk group) | (1, 2, 13) and representative values |
| Degree of risk assortativeness | 0.3 | Informed by infectious disease modeling works | (1, 2, 8) and representative value |
| Relative risk of exposure by risk group |  | Quantitative estimates based on the plausible level of risk of exposure to HCV infection | Based on a power-law function motivated by analyses of the architecture of complex weighted networks (14, 15), and by an analysis of the average separation between individuals in a network or a sub-network (16) |
| First risk group  (lowest risk group)  Second risk group  Third risk group  Fourth risk group  Fifth risk group  (highest risk group including populations such as people who inject drugs) | 1.0 (reference group)  5.6  15.6  32.0  56.0 |  |  |

**Table S2. Impact of the target incidence ≤5 per 100,000 people per year scenario on HCV chronic infection prevalence in 19 countries in the Middle East and North Africa.**

| **Countries** | **HCV chronic infection prevalence in 2022 (%)** | **HCV chronic infection prevalence by 2030**  **(%)** | | **HCV chronic infection prevalence reduction strictly attributed to the treatment program scenario by 2030 (%)** |
| --- | --- | --- | --- | --- |
|  |  | No-treatment intervention scenario | Target incidence ≤5 per 100,000 people per year scenario |  |
| Afghanistan | 0.41 (0.26-0.67) | 0.36 (0.21-0.62) | 0.05 (0.05-0.05) | 87.2 (75.5-93.5) |
| Algeria | 0.10 (0.02-0.49) | 0.08 (0.01-0.42) | 0.05 (0.01-0.05) | 29.6 (0.0-88.6) |
| Iran^*^ | 0.15 (0.08-0.40) | 0.12 (0.06-0.31) | -- | -- |
| Iraq | 0.24 (0.10-0.63) | 0.24 (0.08-0.52) | 0.16 (0.08-0.16) | 18.8 (0.0-72.1) |
| Jordan | 0.16 (0.06-0.50) | 0.14 (0.05-0.49) | 0.13 (0.05-0.14) | 9.8 (0.0-83.1) |
| Kuwait | 0.84 (0.19-2.68) | 0.63 (0.14-2.08) | 0.10 (0.08-0.11) | 84.2 (17.5-96.1) |
| Lebanon^*^ | 0.11 (0.03-0.48) | 0.06 (0.02-0.29) | -- | -- |
| Libya | 0.94 (0.61-1.62) | 0.89 (0.58-1.54) | 0.25 (0.24-0.26) | 72.0 (56.2-84.3) |
| Morocco | 0.40 (0.22-0.70) | 0.29 (0.16-0.52) | 0.10 (0.10-0.11) | 65.1 (34.5-78.6) |
| Oman | 0.34 (0.18-0.63) | 0.23 (0.12-0.45) | 0.03 (0.03-0.03) | 87.4 (74.7-93.8) |
| Palestine^*^ | 0.31 (0.19-0.47) | 0.23 (0.14-0.36) | -- | -- |
| Qatar | 0.37 (0.37-0.44) | 0.34 (0.34-0.41) | 0.09 (0.09-0.09) | 74.9 (74.6-79.1) |
| Saudi Arabia | 0.40 (0.23-0.71) | 0.29 (0.17-0.52) | 0.14 (0.14-0.14) | 53.0 (15.7-74.7) |
| Somalia | 0.24 (0.08-0.66) | 0.19 (0.05-0.56) | 0.08 (0.05-0.09) | 57.9 (0.0-89.6) |
| Sudan | 0.43 (0.14-1.13) | 0.35 (0.11-0.98) | 0.12 (0.11-0.12) | 64.7 (0.0-90.1) |
| Syria^*^ | 0.29 (0.18-0.49) | 0.24 (0.15-0.41) | -- | -- |
| Tunisia^*^ | 0.30 (0.14-0.74) | 0.23 (0.11-0.58) | -- | -- |
| United Arab Emirates | 0.98 (0.52-1.87) | 0.69 (0.36-1.39) | 0.13 (0.13-0.13) | 81.5 (62.9-91.7) |
| Yemen | 0.60 (0.29-1.15) | 0.39 (0.18-0.77) | 0.08 (0.07-0.09) | 78.5 (49.6-90.4) |

^*^No estimates could be derived because the target incidence **≤**5 per 100,000 people per year scenario has been reached without a treatment program.

**Table S3. Impact of the target incidence ≤5 per 100,000 people per year scenario on HCV incidence in 19 countries in the Middle East and North Africa.**

| **Countries** | **Incidence rate in 2022**  **(per 100,000 person-years)** | **Incidence rate by 2030**  **(per 100,000 person-years)** | | **Incidence rate reduction strictly attributed to the treatment program scenario by 2030 (%)** |
| --- | --- | --- | --- | --- |
|  |  | No-treatment intervention scenario | Target incidence ≤5 per 100,000 people per year scenario |  |
| Afghanistan | 49.6 (27.3-91.2) | 37.8 (20.0-72.3) | 5.0 (5.0-5.0) | 86.8 (75.0-93.1) |
| Algeria | 9.8 (1.4-55.3) | 7.1 (0.9-42.8) | 5.0 (0.9-5.0) | 29.4 (0.0-88.3) |
| Iran^*^ | 5.8 (2.9-16.4) | 3.8 (1.9-11.0) | -- | -- |
| Iraq | 8.4 (3.5-27.1) | 6.2 (3.1-20.6) | 5.0 (3.1-5.0) | 18.6 (0.0-76.5) |
| Jordan | 6.1 (1.6-27.1) | 5.5 (1.4-27.4) | 5.0 (1.4-5.0) | 9.5 (0.0-81.8) |
| Kuwait | 53.0 (10.6-197.8) | 31.2 (6.1-123.2) | 5.0 (5.0-5.0) | 84.0 (17.4-95.9) |
| Lebanon^*^ | 5.2 (1.1-32.2) | 3.2 (0.6-23.4) | -- | -- |
| Libya | 19.7 (12.7-34.2) | 17.4 (11.3-30.3) | 5.0 (5.0-5.0) | 71.2 (55.5-83.5) |
| Morocco | 23.6 (12.5-43.3) | 14.7 (7.7-27.3) | 5.0 (5.0-5.5) | 65.9 (34.9-79.7) |
| Oman | 61.1 (31.6-118.5) | 39.0 (19.5-78.3) | 5.0 (5.0-5.0) | 87.2 (74.4-93.6) |
| Palestine^*^ | 4.8 (3.1-8.2) | 3.5 (2.2-6.1) | -- | -- |
| Qatar | 22.5 (22.4-26.9) | 19.7 (19.5-23.6) | 5.0 (5.0-5.0) | 74.6 (74.4-78.8) |
| Saudi Arabia | 16.1 (9.1-29.4) | 10.6 (5.9-19.5) | 5.0 (5.0-5.0) | 52.7 (15.6-74.3) |
| Somalia | 15.5 (3.5-52.8) | 11.7 (2.4-44.8) | 5.0 (2.4-5.0) | 57.2 (0.0-88.8) |
| Sudan | 18.0 (5.6-58.5) | 13.9 (4.1-47.2) | 5.0 (4.1-5.0) | 63.9 (0.0-89.4) |
| Syria^*^ | 4.5 (2.7-7.9) | 3.6 (2.1-6.5) | -- | -- |
| Tunisia^*^ | 3.5 (1.6-9.7) | 2.5 (1.1-7.0) | -- | -- |
| United Arab Emirates | 39.0 (19.8-83.7) | 27.0 (13.5-60.3) | 5.0 (5.0-5.0) | 81.4 (62.8-91.7) |
| Yemen | 42.9 (19.2-90.2) | 22.9 (9.9-50.3) | 5.0 (5.0-5.0) | 78.1 (49.3-90.0) |

^*^No estimates could be derived because the target incidence **≤**5 per 100,000 people per year scenario has been reached without a treatment program.

**Table S4.** **Key program indicators for the target incidence ≤5 per 100,000 per year people scenario in 19 countries in the Middle East and North Africa.**

| **Countries** | **Number of treatments from 2023 up to 2030** | **Treatment coverage by 2030 (%)** | **Number of HCV infections averted by 2030** | **Number of treatments per infection averted by 2030** |
| --- | --- | --- | --- | --- |
| Afghanistan | 173,848  (92,941-306,155) | 81.0  (66.5-89.5) | 80,650  (34,678-173,484) | 2.2  (1.8-2.7) |
| Algeria | 14,449  (0-219,841) | 21.6  (0.0-81.7) | 4,280  (0-96,743) | 3.4  (Omitted^†^) |
| Iran^*^ | -- | -- | -- | -- |
| Iraq | 21,793  (0-50,516) | 16.4  (0.0-25.5) | 2,113  (0-4,132) | 10.3  (Omitted^†^) |
| Jordan | 2,034  (0-52,611) | 7.6  (0.0-76.7) | 237  (0-12,174) | 8.6  (Omitted^†^) |
| Kuwait | 30,631  (1,427-112,990) | 78.7  (13.6-94.1) | 6,446  (209-31,988) | 4.8  (3.5-6.8) |
| Lebanon^*^ | -- | -- | -- | -- |
| Libya | 51,653  (26,593-103,557) | 66.7  (50.1-80.9) | 3,800  (1,805-8,198) | 13.6  (12.6-14.7) |
| Morocco | 115,894  (33,434-251,166) | 53.0  (27.2-66.2) | 22,364  (5,243-64,799) | 5.2  (3.9-6.4) |
| Oman | 14,470  (6,498-29,590) | 76.9  (60.6-87.3) | 9,719  (3,859-22,069) | 1.5  (1.3-1.7) |
| Palestine^*^ | -- | -- | -- | -- |
| Qatar | 9,288  (9,195-11,512) | 67.0  (66.9-72.0) | 2,005  (1,982-2,593) | 4.6  (4.4-4.6) |
| Saudi Arabia | 82,457  (14,470-206,219) | 45.3  (12.5-68.2) | 10,258  (1,532-28,953) | 8.0  (7.1-9.4) |
| Somalia | 28,688  (0-121,585) | 49.1  (0.0-84.6) | 6,199  (0-41,297) | 4.6  (Omitted^†^) |
| Sudan | 151,200  (0-554,942) | 59.0  (0.0-87.3) | 21,895  (0-117,467) | 6.9  (Omitted^†^) |
| Syria^*^ | -- | -- | -- | -- |
| Tunisia^*^ | -- | -- | -- | -- |
| United Arab Emirates | 74,009  (30,271-162,091) | 74.9  (55.4-86.7) | 11,455  (3,965-33,378) | 6.5  (4.9-7.6) |
| Yemen | 170,073  (51,761-385,499) | 71.7  (40.9-85.8) | 39,617  (9,717-107,272) | 4.3  (3.6-5.3) |

^*^No estimates could be derived because the target incidence **≤**5 per 100,000 people per year scenario has been reached without a treatment program.

^†^Uncertainty interval could not be estimated because the target incidence **≤**5 per 100,000 people per year is reached in the uncertainty runs

precluding an estimate of the lower bound.

**References**

1. Ayoub HH, Abu-Raddad LJ. Impact of treatment on hepatitis C virus transmission and incidence in Egypt: A case for treatment as prevention. J Viral Hepat. 2017;24(6):486-95.

2. Ayoub HH, Al Kanaani Z, Abu-Raddad LJ. Characterizing the temporal evolution of the hepatitis C virus epidemic in Pakistan. J Viral Hepat. 2018;25(6):670-9.

3. Ayoub HH, Abu-Raddad LJ. Treatment as prevention for hepatitis C virus in Pakistan: mathematical modelling projections. BMJ open. 2019;9(5):e026600.

4. Akbarzadeh V, Mumtaz GR, Awad SF, Weiss HA, Abu-Raddad LJ. HCV prevalence can predict HIV epidemic potential among people who inject drugs: mathematical modeling analysis. BMC Public Health. 2016;16(1):1216.

5. Vickerman P, Martin NK, Hickman M. Understanding the trends in HIV and hepatitis C prevalence amongst injecting drug users in different settings—implications for intervention impact. Drug and alcohol dependence. 2012;123(1):122-31.

6. Vickerman P, Grebely J, Dore GJ, Sacks-Davis R, Page K, Thomas DL, et al. The more you look, the more you find: effects of hepatitis C virus testing interval on reinfection incidence and clearance and implications for future vaccine study design. J Infect Dis. 2012;205(9):1342-50.

7. Hollingsworth TD, Anderson RM, Fraser C. HIV-1 transmission, by stage of infection. Journal of Infectious Diseases. 2008;198(5):687-93.

8. Abu-Raddad LJ, Longini Jr IM. No HIV stage is dominant in driving the HIV epidemic in sub-Saharan Africa. Aids. 2008;22(9):1055-61.

9. Vickerman P, Hickman M, Judd A. Modelling the impact on Hepatitis C transmission of reducing syringe sharing: London case study. International Journal of Epidemiology. 2007;36(2):396-405.

10. Osburn WO, Fisher BE, Dowd KA, Urban G, Liu L, Ray SC, et al. Spontaneous control of primary hepatitis C virus infection and immunity against persistent reinfection. Gastroenterology. 2010;138(1):315-24.

11. Grebely J, Page K, Sacks‐Davis R, Loeff MS, Rice TM, Bruneau J, et al. The effects of female sex, viral genotype, and IL28B genotype on spontaneous clearance of acute hepatitis C virus infection. Hepatology. 2014;59(1):109-20.

12. Grebely J, Prins M, Hellard M, Cox AL, Osburn WO, Lauer G, et al. Hepatitis C virus clearance, reinfection, and persistence, with insights from studies of injecting drug users: towards a vaccine. Lancet Infect Dis. 2012;12(5):408-14.

13. Mumtaz GR, Weiss HA, Thomas SL, Riome S, Setayesh H, Riedner G, et al. HIV among people who inject drugs in the Middle East and North Africa: systematic review and data synthesis. PLoS Med. 2014;11(6):e1001663.

14. Barendregt JJ, Van Oortmarssen GJ, Vos T, Murray CJ. A generic model for the assessment of disease epidemiology: the computational basis of DisMod II. Popul Health Metr. 2003;1(1):4.

15. Barrat A, Barthelemy M, Pastor-Satorras R, Vespignani A. The architecture of complex weighted networks. Proceedings of the National Academy of Sciences of the United States of America. 2004;101(11):3747-52.

16. Barabasi A-L. Linked: How everything is connected to everything else and what it means. Plume Editors. 2002.
